# Sexual selection accelerates signal evolution during speciation in birds

**DOI:** 10.1098/rspb.2013.1065

**Published:** 2013-09-07

**Authors:** Nathalie Seddon, Carlos A. Botero, Joseph A. Tobias, Peter O. Dunn, Hannah E. A. MacGregor, Dustin R. Rubenstein, J. Albert C. Uy, Jason T. Weir, Linda A. Whittingham, Rebecca J. Safran

**Affiliations:** 1 Edward Grey Institute, University of Oxford, South Parks Road, Oxford OX1 3PS, UK; 2 Initiative for Biological Complexity, Southeast Climate Science Center North Carolina State University, 3510 Thomas Hall, Raleigh, NC 27695, USA; 3 Southeast Climate Science Center North Carolina State University, 3510 Thomas Hall, Raleigh, NC 27695, USA; 4 Behavioral and Molecular Ecology Group, Department of Biological Sciences, University of Wisconsin, Milwaukee, WI 53201, USA; 5 Department of Ecology, Evolution and Environmental Biology, Columbia University, New York, NY 10027, USA; 6 Department of Biology, University of Miami, Coral Gables, FL 33146, USA; 7 Department of Biological Sciences, University of Toronto, Ontario, CanadaM1C 1A4; 8 Department of Ecology and Evolutionary Biology, University of Colorado, Boulder, CO 80309, USA

**Keywords:** comparative analyses, plumage dichromatism, evolutionary rates, sexual selection, sister species, speciation

## Abstract

Sexual selection is proposed to be an important driver of diversification in animal systems, yet previous tests of this hypothesis have produced mixed results and the mechanisms involved remain unclear. Here, we use a novel phylogenetic approach to assess the influence of sexual selection on patterns of evolutionary change during 84 recent speciation events across 23 passerine bird families. We show that elevated levels of sexual selection are associated with more rapid phenotypic divergence between related lineages, and that this effect is restricted to male plumage traits proposed to function in mate choice and species recognition. Conversely, we found no evidence that sexual selection promoted divergence in female plumage traits, or in male traits related to foraging and locomotion. These results provide strong evidence that female choice and male–male competition are dominant mechanisms driving divergence during speciation in birds, potentially linking sexual selection to the accelerated evolution of pre-mating reproductive isolation.

## 1. Introduction

The role of sexual signals in maintaining reproductive isolation between lineages has long been recognized [1], leading to the explicit proposal that sexual selection—that is, selection within and between the sexes, driven by competition for matings—is a powerful evolutionary force promoting speciation [2,3]. This hypothesis is well supported by theoretical models, particularly those focusing on the evolution of reproductive isolation in sympatry [4,5]. However, it has proved difficult to test experimentally [6], and thus remains a contentious area in speciation research [7]. In particular, the extent to which sexual selection helps to explain variation in rates of either phenotypic divergence or lineage diversification remains unclear [8,9].

Most empirical support for sexual selection as a driver of speciation comes from comparative analyses, particularly those showing correlations between species richness and various estimates of sexual selection [10,11]. Yet, such correlations are by no means universal [12,13] and suffer from at least two methodological drawbacks. First, species richness may be underestimated in groups lacking sexual ornamentation simply because cryptic species are more readily overlooked by taxonomists [9]. Second, the number of extant species in a clade is not only the product of speciation but also of extinction. The contribution of extinction is generally unknown, but theoretically increases at larger taxonomic scales, perhaps explaining why studies comparing across distantly related taxa yield weak associations between sexual selection and speciation [8,9,13]. In addition, comparative analyses based on species richness tell us little about causal mechanisms linking sexual selection to diversification as the same patterns can be explained by sexual conflict, reinforcement or faster evolution of sexual signals via mate choice and intrasexual competition [9,14].

An alternative approach has been to focus explicitly on testing the mechanism linking sexual selection to speciation. For example, many studies provide evidence that closely related or incipient species differ primarily in male traits used in courtship [15,16], suggesting that the key effect of sexual selection lies in the divergence of sexual signals. However, these findings are typically based on a small sample of species and do not rule out the possibility that sexual trait divergence is mainly caused by ecological selection (reviewed in [17,18]). This can occur because of adaptation to differences in the signal transmission properties of habitats—that is, ‘sensory drive’ [19]—or because ecological selection shapes phenotypes involved in signal production—that is, ‘magic traits’ [20]—two processes known to be widespread in animals [18,21].

Recent mechanistic studies have attempted to disentangle sexual from ecological selection by using phylogenetic techniques to examine rates of trait evolution over time. Some [22] have revealed that sexual signals evolve faster than ecological traits across single clades. While this result fortifies the argument that sexual selection contributes to diversification by increasing rates of signal evolution, it provides only partial support. One reason is that evolutionary rates calculated across entire clades may be confounded by gradual (i.e. anagenetic) evolutionary change within lineages. Phenotypic divergence may thus be exaggerated in older lineages, and thus across deeper nodes with little relevance to speciation. Another reason is that hidden nodes associated with extinction events may be biased towards deeper timescales, particularly in older clades, potentially distorting rates of evolution [23]. The most obvious solution is to apply similar phylogenetic techniques to a broad sample of recent speciation events, thus minimizing the effect of longer term processes and maximizing the relevance to diversification [24]. However, this approach has not yet been attempted in conjunction with indices of sexual selection.

The aim of this study was to integrate the broad sampling of comparative techniques with the targeted approach of mechanistic studies in order to test the hypothesis that sexual selection promotes speciation by accelerating the evolutionary divergence of sexual signals between closely related taxa. To achieve this, we examined the relationship between an indicator of sexual selection and the degree of phenotypic divergence across 84 pairs of closely related bird species from 23 passerine families. We focused on birds because of the general availability of phylogenetic and phenotypic data, and because avian plumage dichromatism is a robust, well-established and widely used index of sexual selection [8]. Using mitochondrial DNA (mtDNA) sequence divergence to estimate the timing of speciation events, we tested the predictions that sexual selection: (1) increases the extent of phenotypic divergence between closely related species over time, (2) produces contrasting rates of trait divergence between males and females, and (3) elevates the rate at which new species form.

If the formation of new species is facilitated by the acceleration of trait divergence under sexual selection (prediction 1), we expect sexual selection to be associated with faster accumulation of phenotypic differences between closely related species. Moreover, this process should be accentuated in traits associated with mate choice or intrasexual competition, as reproductive isolation is theoretically achieved by the evolution of morphological or behavioural differences between lineages that reduce hybrid viability, decrease mating success or prevent mating altogether [25]. We further predict that rates of trait divergence should be either sex-specific or at least stronger in males (prediction 2). Although this prediction follows logically from the generality that sexual selection acts predominantly on males [26], we are not aware of any previous comparative tests, and the extent to which sexual selection generates sex-specific rates of trait evolution across taxa remains unknown. The third prediction, that pairs of species experiencing more intense sexual selection are associated with more recent divergence times, tests the idea that sexual selection increases the frequency with which new species form.

## 2. Material and methods

### (a) Study species

We used published molecular phylogenies to select a sample of recently diverged species pairs from approximately 1000 bird species with data on sex-specific plumage reflectance measured with a spectrophotometer [27]. To focus on recently diverged lineages, we identified species pairs that were each other's closest relative (i.e. sister species). In our first, but not in our second or third analysis (see below), we assumed that phenotypic divergence between closely related congeners was informative about rates of phenotypic divergence during recent evolutionary history, even when lineages were not true sisters. Thus, we generated further species comparisons (hereafter ‘clade sisters’) by pairing a single lineage (a ‘focal species’) with a member of a small (less than or equal to 5 species) sister clade. Both types of species pairs were identified from published phylogenetic trees and determined by the availability of molecular data. Overall, our sample of species comparisons contained 84 species pairs, including 39 sister species and 45 clade sisters widely but nonetheless randomly distributed across the passerine radiation (23 families). For full details, see the electronic supplementary material, appendices S1 and S2.

### (b) Strengths of the sister-species approach

By measuring phenotypic divergence among sister species and clade sisters, we restricted our dataset to the branching tips of phylogenies and thus effectively focused on recent speciation events. This approach deals with a range of factors that potentially confound clade-wide analyses, which instead extract information from a greater span of evolutionary time. First, although it is possible that lineages go extinct shortly after becoming reproductively isolated, the potential bias introduced by extinction is greater over deeper nodes, simply because extinction accumulates over time [28]. Second, we selected pairs of lineages on the basis of genetic data from well-sampled phylogenies, often including intraspecific lineages. This reduces the likelihood of overlooking cryptic species, in comparison with current taxonomy based on morphological traits. In addition, the problem of cryptic species is potentially far greater for studies focusing on species richness and other clade-wide patterns, as errors again accumulate across clades. Third, our approach helps to control for the influence of ecological adaptation in driving gradual phenotypic divergence, as sister species are relatively young and tend to occur in similar habitats because niches are phylogenetically conserved [29].

### (c) Quantifying phenotype

We quantified two components of avian phenotype: morphological traits and plumage traits (see the electronic supplementary material, table S1). The term ‘morphological traits’ refers to phenotypic attributes linked to aspects of the foraging niche and locomotion. By contrast, we use the term ‘plumage traits’ to refer to the colour of feathers, an attribute that plays no role in foraging or locomotion, but instead is proposed to function as a visual signal. Morphological traits are quantified in terms of biometric measurements; plumage traits are quantified in terms of spectral reflectance.

#### (i) Morphological traits

Beak, tarsus and wing length were measured from three male and three female adult specimens, where possible, for 69 pairs of species (see the electronic supplementary material, appendix S2).

#### (ii) Plumage traits

We did not score plumage differences using human vision because spectral sensitivity in humans differs from that of birds, particularly in the ultraviolet (UV) spectrum [30]. Instead, we used a spectrophotometer to quantify plumage reflectance from 320 to 700 nm. Spectrophotometric measurements were taken from six body regions (crown, throat, belly, tail, back and wing coverts) of three male and three female adult specimens in breeding plumage for 84 pairs of species. We used principal components (PCs) analysis to collapse reflectance data into a reduced set of independent axes summarizing spectral variation. The resulting two PC were indices of chroma and hue, independent of brightness: PC1 was positively correlated with reflectance in the 400–480 nm range (i.e. short wavelength (SW) chroma); and PC2 was positively correlated with reflectance in 320–380 nm range (i.e. UV reflectance; electronic supplementary material, table S2). For SW chroma and UV reflectance, we calculated the average for males and females of each species for each body region. For full details, see the electronic supplementary material, appendix S1.

### (d) Estimating extent of phenotypic divergence

To compare the extent of divergence in different phenotypic traits (see the electronic supplementary material, table S1), we computed a standardized score (*z*) for each trait value, where *z* = (trait value for a given species − mean trait value across all species)/(standard deviation of trait value across all species). For each sex and for each trait, we computed divergence as the Euclidean distance of the absolute value of the difference in *z*-scores between pairs of sister species. Because some avian sisters differ dramatically in a single phenotypic character, whereas others differ moderately in multiple characters, we estimated phenotypic divergence between each pair of lineages in two ways: (i) *maximum* extent of phenotypic divergence (the largest *z*-score for any morphological or plumage trait), and (ii) *total* extent of phenotypic divergence (the sum of the *z*-scores across all traits). We note that rates of change in plumage colour were calculated *between species*, whereas plumage dichromatism was calculated from sex-differences in plumage colour *within species*.

### (e) Index of sexual selection

Avian plumage dichromatism—typically characterized by males possessing a brighter, more distinctive or more colourful phenotype than females—has long been assumed to arise primarily from female choice or male–male competition [26]. Although alternative mechanisms have been proposed, numerous studies have revealed strong positive relationships between dichromatism and other indices of sexual selection such as testes size, the degree of polygyny and the frequency of extra-pair paternity [31]. Dichromatism is therefore the most robust index currently available for broad-scale comparative studies and has been widely used to test the effects of sexual selection in birds [10–12,32] and other organisms (reviewed in [8]).

A variety of methods to calculate plumage dichromatism are available, some of which model the spectral sensitivity of the avian eye, although different approaches tend to yield highly correlated estimates regardless of whether receiver perception is taken into account [27]. For simplicity, and to avoid making assumptions about colour perception in a range of species for which data on spectral sensitivity are lacking, we calculated sex-differences directly from spectrophotometric analyses of plumage. Plumage dichromatism was quantified for each species as the mean Euclidean distance between males and females for each of the two PCs derived from plumage reflectance data (see the electronic supplementary material, table S2) at each of the six body regions. We then summed the differences between males and females for each PC across all body regions to produce the overall dichromatism score for a species. A dichromatism score of zero indicates identical coloration in both sexes (monochromatism) with higher positive values indicating greater degrees of dichromatism. In the models presented below, we use the average of the sexual dichromatism scores of both species in each pair as an index of the strength of sexual selection during speciation.

### (f) Additional predictors of phenotypic divergence

To explore the role of other factors known to influence estimates of phenotypic divergence in birds, including allometric effects, we collected the following additional data.

#### (i) Evolutionary age

It is important to consider estimates of trait divergence in the context of evolutionary time, so we used mtDNA sequence divergence and a standard molecular clock approach to calculate the evolutionary age of each lineage pair. All cyt-*b* sequences were downloaded from GenBank for 138 species and aligned in MEGA v. 5.0 (accession numbers are reported in the electronic supplementary material, table S1). Where a choice of sequences was available for a given species, we chose the longest. The final alignments of cyt-*b* were then concatenated in R v. 2.13.0. The phylogenetic tree (see the electronic supplementary material, figure S1) was reconstructed using a relaxed clock approach in BEAST v. 1.6.1 [33], and the model search was restricted using topological constraints defining *a priori* all the known species pairs and genera in our sample. Parameters for codon positions (1 + 2) and 3 were calculated separately, using a GTR + G model of sequence evolution. We estimated the approximate age of pairs of lineages (i.e. time from the present to the most recent common ancestor) by applying a molecular clock of 1.05% per lineage per million years to GTR-g genetic distances of cyt-*b* sequences using PAUP v. 4.0b10 [34]. The 1.05% clock is based on 74 avian calibrations spanning 12 taxonomic orders using the same gene and model of sequence evolution as used here [35].The clock is consistent over the past 12 Myr and across most avian orders, supporting its use for estimating evolutionary age over the timescales relevant to this study. Moreover, for birds, there are similar rates of sequence evolution across the latitudinal gradient in mitochondrial protein-coding genes [35,36].

#### (ii) Geographical relationships

Divergence in morphological traits or signals between closely related species may be accelerated by species interactions (e.g. character displacement; [37,38]). To examine the role of such interactions in driving phenotypic divergence in our sample, we categorized species pairs as either allopatric (no geographical contact between pair members during the breeding season) or sympatric (breeding ranges overlapping). Data for most species pairs were extracted from published studies (see the electronic supplementary material, appendix S1). For additional species pairs, we categorized geographical relationships of species with methods following Weir & Price [39], based on high-quality range maps from recent sources (see the electronic supplementary material, appendix S2). Allopatry or sympatry was included as a binary fixed effect in models of trait divergence (see below).

#### (iii) Body mass

To control for allometric effects, we included mean body mass of the species pair (averaged across the sexes if data for both were provided) as a covariate in our mixed models (see the electronic supplementary material, appendix S1).

### (g) Analytical approaches

#### (i) Analysis 1: effect of sexual selection on extent of phenotypic divergence

We used linear mixed effect models (LMMs) to investigate whether the extent of phenotypic divergence among closely related lineages varies according to levels of sexual selection (prediction 1), without making any *a priori* assumptions about the ultimate function of phenotypic traits. We modelled the maximum and total extent of phenotypic divergence (dependent variables) in relation to several predictors: an index of sexual selection (mean value of sexual dichromatism within a pair), sex and the interaction between sex and dichromatism. The variable ‘sex’ was included as a factor in the models to assess whether trait divergence between species was more pronounced in males (prediction 2). We controlled for phylogenetic inertia using two complementary techniques: first by including Family and Genus as nested random effects in our LMMs, and then by conducting phylogenetic generalized least-squares regression (PGLS; [40]). The LMMs are robust to analysis of repeated measures and can therefore be used on all unique species pairs in our dataset (*n* = 69); however, they assume that the phylogenetic signal of phenotypic divergence is weak or non-existent (i.e. lambda (*λ*) approaches 0). By contrast, although PGLS models can only be run on a smaller subset of independent species pairs (*n* = 52), they make no assumptions about *λ*, and instead estimate *λ* directly from the maximum-likelihood tree (see the electronic supplementary material, figure S1).

Mutual mate-choice and intrasexual aggression in both sexes can result in males and females sharing equally bright plumage or elaborate ornaments [41]. The widespread occurrence of this form of ‘mutual ornamentation’ in birds may potentially yield low dichromatism scores in species with high levels of inter- and/or intrasexual selection. To assess the effect of including such species in our analyses, we re-ran PGLS models excluding mutually ornamented species. We defined mutual ornamentation as the occurrence of a similar extent of highly colourful or iridescent plumage patches, or bold patterning such as stripes or spots, in both males and females (see the electronic supplementary material, appendix S1). These models retained species with dull monomorphism, that is, those in which males and females shared similar plumage features but lacked striking colours or ornamentation.

#### (ii) Analysis 2: effect of sexual selection on evolutionary rates of phenotypic divergence

We compared the fit of Brownian motion (BM) and Ornstein–Uhlenbeck (OU) models with a constant rate of evolution, *β*, with that of BM and OU models in which *β* is allowed to vary linearly with increased intensity of sexual dichromatism, *S*. The OU model has an evolutionary constraint parameter, *α*, which was also allowed to vary linearly with increased *S.* These models estimate the rate of evolutionary divergence that is most likely to produce the within-pair differences observed in the data, and thus provide a complementary approach to determining whether sexual selection elevates rates of phenotypic divergence (prediction 1). We also used this approach to estimate the rate of divergence of each phenotypic trait in males, females and both sexes combined, to assess differences between the sexes (prediction 2). To test whether sexual dichromatism increases rates of divergence in a given trait, we compared the likelihood fits of the two model types using likelihood functions given in Weir & Wheatcroft [24]:
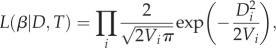
where *D* is the Euclidean distance between species pairs, *T* is the genetic distance separating each pair, and
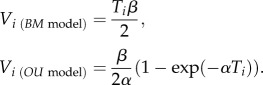
In models with sexual dichromatism, both *α* and *β* were assumed to be linear functions of *S*, such that *β*_(with sexual dichromatism)_ = *b*_*β*_*S*_*i*_ + *c*_*β*_, and *α*_(with sexual dichromatism)_ = *b*_*α*_*S*_*i*_ + *c*_*α*_ and (where *b*_*β*/*α*_ and *c*_*β*/*α*_ are the slope and intercept parameters describing the linear change of *β* and *α* as a function of *S*). As *α* approaches 0, the model collapses to BM. We used Akaike criterion with small sample correction (AICc) and Akaike weights to assess support for models with or without sexual dichromatism included. To maximize independence of species pairs, we only used non-phylogenetically nested pairs in this analysis, retaining from our dataset only the youngest pairs in cases where the same species was present in two or more pairs.

#### (iii) Analysis 3: effect of sexual dichromatism on diversification

To assess whether sexual selection drives faster rates of cladogenesis and extinction (prediction 3), we applied contrasting birth–death models to our data. In the first model, a single set of speciation and extinction rates was estimated for all 39 pairs of true sister species in our dataset. In the second model, rates of cladogenesis and extinction were allowed to vary linearly with increasing intensity of sexual dichromatism. Briefly, these models estimate the rates of speciation and extinction that would most probably yield the distribution of species’ ages that we see in our sample. The likelihood functions for these models follow Weir & Schluter [42]:
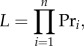
where Pr*_i_* is the probability that species *i* was drawn from a probability distribution of sister-species ages simulated under a birth–death model with species origination rate *λ*, extinction rate *μ* and a lag-time to species recognition *φ* (see the electronic supplementary material, appendix S1). The lag-time correction prunes out nodes from phylogenetic trees if they post-date a lag-time drawn at random from an exponential distribution with mean age *φ*. This is intended to correct for the fact that empirical species trees lack nodes representing intraspecific splits between taxa currently recognized as subspecies. The correction allows direct comparison between sister-species’ ages from simulated and empirical trees which would otherwise not be possible [42]. We compared a two-parameter model in which all sister species had a single rate of *λ* and *μ* with a model in which *λ* and *μ* changed linearly with increasing sexual dichromatism (a four-parameter model with two slopes and two intercepts). Both models estimated a single rate of *φ* (i.e. one additional parameter for both models), which for simplicity was assumed not to vary with increasing sexual dichromatism.

Models used in analyses 2 and 3 were implemented through custom-made routines written by J.T.W. in R and submitted to GEIGER (see the electronic supplementary material, appendix S1).

## 3. Results

### (a) Effect of sexual selection on extent of phenotypic divergence

**Figure 1.**
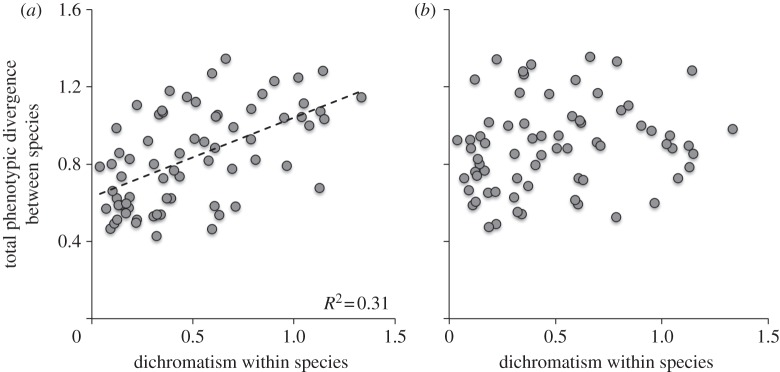
Relationship between the total extent of phenotypic divergence between species (total divergence summed across 15 traits, log-transformed) and intensity of sexual selection (log-transformed dichromatism within species, averaged for the two members of each species pair). Data are shown for (*a*) males and (*b*) females (*n* = 69 species pairs).

Our mixed models revealed that plumage dichromatism had a strong effect on total phenotypic divergence between species, when pooling morphological and plumage traits (table 1). They also showed that both total and maximum phenotypic divergence between species were predicted by a highly significant interaction between sex and dichromatism (table 1), caused by a positive effect of dichromatism in males (figure 1*a*), but not in females (figure 1*b*). The effect of the interaction between sex and dichromatism on phenotypic divergence remained strong when controlling for the phylogenetic signal of phenotypic divergence using PGLS models (see the electronic supplementary material, table S3) and after removing species with mutual ornamentation (see the electronic supplementary material, table S4). Moreover, alternative factors, such as evolutionary age, body mass and sympatry, showed no effects on total or maximum phenotypic divergence in any model.

**Table 1.** Linear mixed effect models of (*a*) total and (*b*) maximum phenotypic divergence between closely related species in relation to the intensity of sexual selection within species (dichromatism), sex and other potentially confounding variables (*n* = 69 pairs).

fixed effects	parameter estimate (*β*)	s.e.	d.f.	*t*	*p*
(*a*)					
dichromatism	4.36	1.44	44.70	3.03	0.004
sex	−0.49	0.14	67.00	3.37	0.001
dichromatism × sex	2.39	0.43	67.00	−5.53	<0.0001
evolutionary age	0.51	0.60	67.00	0.85	0.40
sympatry	0.24	0.52	56.14	0.45	0.65
body mass	2.78	1.42	44.49	1.96	0.06
random terms	variance component	s.e.	d.f.	LRT	*p*
sisterhood	10.40	10.91	1	1.69	0.19
species 1	−3.61	9.26	1	0.07	0.79
species 2	−1.07	5.47	1	0.04	0.84
family (genus)	4.49	2.26	1	7.23	0.007
residual variance	2.89	0.50			
fixed effects	parameter estimate (*β*)	s.e.	d.f.	*t*	*p*
(*b*)					
dichromatism	0.57	0.32	50.17	1.79	0.079
sex	−0.08	0.04	67.00	1.83	0.071
dichromatism × sex	0.42	0.13	67.00	−3.33	0.001
evolutionary age	−0.00	0.13	67.00	−0.00	0.99
sympatry	−0.05	0.12	60.01	−0.46	0.65
body mass	0.11	0.2	38.49	0.31	0.58
random terms	variance component	s.e.	d.f.	LRT	*p*
sisterhood	−0.20	0.28	1	0.30	0.58
species 1	0.29	0.16	1	1.22	0.27
species 2	0.23	0.25	1	0.38	0.54
family (genus)	0.16	0.11	1	4.10	0.04
residual variance	0.25	0.04

### (b) Effect of sexual selection on evolutionary rates of phenotypic divergence

**Figure 2.**
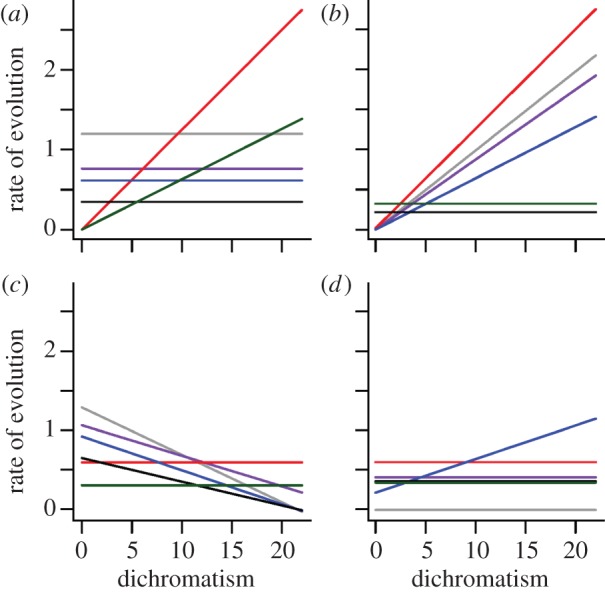
Estimated rates of plumage colour evolution in (*a*,*b*) male and (*c*,*d*) female birds as a function of sexual selection (dichromatism). For illustration purposes, the rates depicted in these plots are from models based on a BM model of evolution (OU model results are difficult to display graphically owing to variation in the constraint parameter, *α*). For (*a*,*c*) SW chroma and (*b*,*d*) UV reflectance: dark grey, back; purple, belly; red, throat; blue, crown; black, tail; green, wing coverts. Both these wavelength categories are visible to birds, but only the former is visible to humans.

Evolutionary models provided a more direct test of the role of sexual selection in driving phenotypic evolution and provided additional insights regarding the interaction between trait function and patterns of phenotypic divergence. They showed that the effect of sexual dichromatism on the rate of phenotypic divergence is not distributed equally among the different male traits (table 2 and figure 2). On the one hand, dichromatism was strongly related to divergence in the colour (SW chroma) and/or amount of UV reflectance from the crown, throat, belly, back and wing (see table 2 and the electronic supplementary material, table S5). For all these traits, we found that the estimated effect of dichromatism (i.e. the ‘slope for rate’ parameter in the electronic supplementary material, table S5) was positive, indicating that evolutionary rates of divergence are accelerated by sexual selection. On the other hand, for the remaining male traits (beak length, tarsus length, wing-chord length and tail colour), we found that the best-fit models did not include dichromatism (see the electronic supplementary material, table S5).

**Table 2.** *Δ*AICc scores showing support for models in which the rate of evolutionary divergence in (*a*) male and (*b*) female traits is assumed to be independent or linearly associated with the strength of sexual selection (SS). (Asterisks denote the best-fit model, that is, where Akaike weight greater than 70% and *Δ*AIC_c_ > 2 when compared with the next best-supported model in the set (details in the electronic supplementary material, table S5). SW, short-wave chroma; UV, ultraviolet reflectance (see main text).)

trait	BM models		OU models	
	excluding SS	including SS	excluding SS	including SS
(*a*)				
beak length	9.11	0.50	0.66	0*
tarsus length	0*	1.73	1.60	5.77
wing length	0*	1.91	2.16	6.13
crown SW	31.87	32.96	2.07	0*
crown UV	45.56	19.07	10.56	0*
throat SW	15.56	11.06	4.67	0*
throat UV	38.05	27.02	2.37	0*
back SW	57.20	59.31	13.69	0*
back UV	51.82	36.81	9.46	0*
belly SW	41.27	42.89	6.34	0*
belly UV	28.91	19.73	11.36	0*
tail SW	13.41	15.26	0*	3.36
tail UV	5.36	7.06	0*	4.30
wing SW	24.76	3.77	18.99	0*
wing UV	3.50	3.88	0*	2.35
(*b*)				
beak	7.56	8.94	0.29	0*
tarsus	4.34	5.27	0*	3.48
wing	3.02	0*	2.20	3.70
crown SW	41.58	36.81	0*	3.31
crown UV	16.40	15.48	0*	0.74
throat SW	7.27	9.29	1.03	0*
throat UV	18.68	20.70	0*	2.58
back SW	77.50	79.67	0*	3.60
back UV	55.99	58.14	0*	1.77
belly SW	47.17	47.02	0*	0.42
belly UV	26.56	26.84	0*	1.98
tail SW	11.47	13.49	0*	2.29
tail UV	16.46	17.91	0*	3.25
wing SW	12.70	14.85	0*	3.23
wing UV	23.88	23.97	0*	3.96

When we focused the same analyses on females, a strikingly different pattern emerged (figure 2). Specifically, we found that the best-fit models did not include dichromatism for any trait (see table 2 and the electronic supplementary material, table S6), thus indicating that more intense sexual selection has little effect on evolutionary rates of phenotypic divergence in females. To determine whether the apparent differences between sexes were supported by the data, we compared models in which a single rate of evolution was estimated for combined data from both sexes with models in which rates were estimated for each sex separately. As observed in analysis 1, the results of this model comparison confirmed significant sexual differences in the tempo of phenotypic divergence (see the electronic supplementary material, table S7).

### (c) Effect of sexual dichromatism on diversification

**Figure 3.**
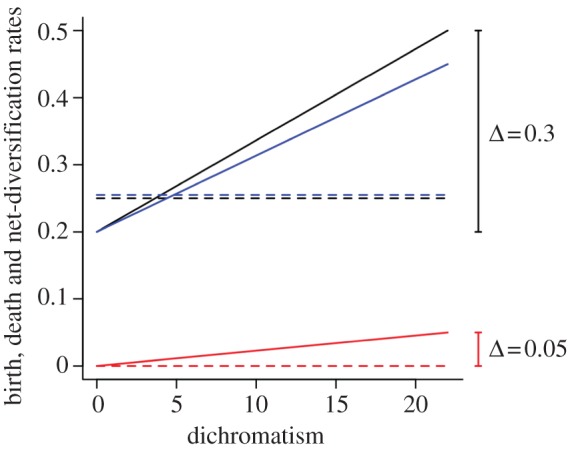
Estimated rates of cladogenesis (*λ*, black), extinction (*μ*, red) and net diversification (blue) as a function of sexual dichromatism. Solid line, variable rates model (*λ* and *μ* change linearly with increasing sexual dichromatism); dashed line, constant rates model (single rate of *λ* and *μ*, see main text). Analyses were restricted to true sister species (*n* = 39 species pairs).

We found that net diversification rates were consistently higher, by a factor of 0.014 per unit increase in dichromatism, in lineages exposed to stronger sexual selection (see the electronic supplementary material, table S8; figure 3). However, the model with variable rates provided only a marginally better fit than the model in which rates of cladogenesis and extinction were assumed to be constant across the sexual selection gradient (*Δ*AIC = 1.47; electronic supplementary material, table S8).

## 4. Discussion

By focusing on multiple speciation events in birds and quantifying phenotypic divergence in the context of evolutionary time, we have tested the role of sexual selection in driving phenotypic evolution during recent speciation events. Our key finding is that the degree of sexual dichromatism was strongly positively associated with the rate at which males of related lineages diverge in the colour of five plumage traits: crown, throat, back, belly and wing-coverts (i.e. shoulders or ‘epaulets’). In accordance with our first prediction, these plumage patches often function as inter- or intrasexual signals (reviewed in [26]). Indeed, empirical studies testing the function of ornamental traits have most often identified the colour of the crown [43], throat [44,45] or wing-coverts [46] as the dominant signals mediating mate-choice and/or intrasexual aggression in birds.

By contrast, we found no support for an effect of sexual selection on rates of sister-species divergence in a suite of male traits that are more closely linked to ecological rather than signalling functions. These include beak length, which is strongly related to foraging and dietary niche [47]; tarsus length, which is tightly linked to foraging substrate [48]; and wing length, which is typically associated with foraging behaviour and dispersal [49]. Moreover, the only plumage trait for which rates of divergence were similarly unconnected to sexual selection was the colour of the tail. Although tail colour of male birds is involved in mate-choice and species recognition in some lineages, it appears to be more important for crypsis and predator avoidance in the majority of avian species [50]. Our results are therefore consistent with the idea that sexual selection has little effect on primarily ecological (i.e. non-signalling) traits, but that it accelerates evolutionary divergence in signals known to mediate reproductive isolation and species recognition.

Additional evidence supporting the role of sexual selection as a driver of species differences is provided by clear sex-specific relationships between sexual dichromatism and phenotypic divergence (prediction 2). Although male signalling traits diverge more rapidly during speciation in highly dichromatic lineages, female trait divergence showed no significant association with dichromatism. Thus, sexual selection appears to accelerate the evolution of key traits in males, but has negligible effects on phenotypic divergence in females, in line with general theories of sex differences in the strength and targets of sexual selection [41,51].

One possible explanation for these contrasting patterns of divergence is that ornamentation in female birds is often associated with mutual mate choice and intrasexual competition among females, factors that tend to promote mutual ornamentation rather than plumage dichromatism [2,41]. Dichromatism may therefore capture variation in sexual selection less accurately in females than in males, reducing the link between dichromatism and phenotypic divergence. Despite this issue, it seems highly likely that pairs of species with high dichromatism scores are currently experiencing strong sexual selection, or did so recently in their evolutionary history [26]. Moreover, dichromatism may only provide a lower-bound estimate of the overall intensity of sexual selection because of potential trade-offs between signalling modalities. Bird species such as the nightingale *Luscinia megarhynchos*, for example, may be monomorphic in plumage yet experience strong sexual selection manifested in elaborate male acoustic signals. Indeed, it has long been hypothesized that investment in one signalling modality constrains investment in another [1], perhaps explaining why pairs of avian sister lineages with low levels of plumage divergence tend to have high levels of song divergence [52]. If such a trade-off exists more broadly in birds, the association between dichromatism and sexual selection in males may be weakened, and the effect of sexual selection on the evolution of male mating signals may be stronger than implied by the coefficients reported here (see the electronic supplementary material, tables S3–S8).

This study is not the first to demonstrate a positive relationship between sexual selection and rates of phenotypic evolution in males. For example, accelerated evolution of male genitalia in insects is driven by mechanisms rooted in sexual conflict and antagonistic coevolution, potentially causing speciation as a result of mating incompatibility (reviewed in [9]). However, our analyses provide, to our knowledge the first comparative evidence that sexual selection consistently promotes rapid evolution of male visual signals, with implications for pre-mating isolation among related lineages. This finding makes sense from a mechanistic perspective for two inter-related reasons. First, species-specific male plumage signals are under selection from female choice and male–male competition, the two primary mechanisms of sexual selection first identified by Darwin [1]. Second, rapid evolution has been demonstrated in the sex (Z) chromosome in birds [53], which is also the only known location of genes coding for both male plumage ornaments and associated female preferences [54].

It is possible that our results are relevant exclusively to birds, a group for which visual signals are particularly important for species recognition and mate-choice, and thus central to pre-mating isolation [55]. However, even in groups with less pronounced secondary sexual characteristics, including mammals [56], fishes [22] and insects [16], mate choice is mediated by a wide range of functionally equivalent signals (e.g. display behaviours, electric pulses and olfactory cues). Given that sexual selection may promote divergence in any such mating signal, the patterns we detect in birds may play out far more generally across animal groups.

The key implication is that sexual selection on male signals may accelerate the evolution of reproductive isolation (prediction 3). In accordance with this idea, the results of our final analysis show, to our knowledge, for the first time, a consistent positive relationship between sexual selection and net rates of diversification (i.e. speciation minus extinction). These rates more than doubled across the sexual dichromatism gradient, supporting the view that cladogenesis and extinction are both likely to be promoted by sexual selection [9,57]. However, we note that constant and variable-rate models in analysis 3 received similar support, indicating that differences in rates were not statistically significant. Thus, although the trend is clear, it is not yet possible to determine whether sexual selection influences rates of diversification in birds, perhaps because the overall effects are weak or simply hard to detect at the current sampling level (data from only 39 pairs of species were available for this test). Further studies based on additional sampling of sister species are required to resolve this issue.

Taken together, our findings provide compelling evidence that sexual selection accelerates the evolution of male plumage traits, particularly in signals widely known to mediate inter- and intrasexual selection. We conclude that female choice and male–male competition are the dominant mechanisms regulating the tempo of phenotypic divergence in key traits involved in pre-mating isolation, thereby influencing the probability that previously allopatric populations merge after secondary contact. Thus, our results help to explain the associations between sexual dichromatism and species richness detected in numerous studies across a range of taxa, and shed further light on the mechanisms by which sexual selection can shape broad-scale patterns of species richness and phenotypic diversity.
